# Invasive ductal breast carcinoma preceded by CALR‐positive essential thrombocythemia

**DOI:** 10.1002/ccr3.3892

**Published:** 2021-02-09

**Authors:** Elrazi A. Ali, Hind Elmalik, Nabil E. Omar, Mohamed A. Yassin

**Affiliations:** ^1^ Internal Medicine Department Hamad Medical Corporation Doha Qatar; ^2^ Medical Oncology and Hematology Department Hamad Medical Corporation Doha Qatar; ^3^ Pharmacy Department National Center for Cancer Care and Research Hamad Medical Corporation Doha Qatar

**Keywords:** breast cancer, calreticulin, essential thrombocythemia, invasive ductal carcinoma, thrombocythemia

## Abstract

Persistent thrombocytosis in patients with cancer needs workup because it can be linked to essential thrombocytosis. The management should be individualized to start treatment for low‐risk essential thrombocytosis due to the combined risk of thrombosis.

## Graphical abstract

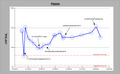

## 1 INTRODUCTION

Thrombocytosis can be a paraneoplastic feature linked to different types of solid tumors. However, it is an unusual finding in patients with breast cancer and has an unfavorable prognosis. Thrombocytosis caused by essential thrombocythemia (ET) is not known to be linked to breast cancer. We report a patient diagnosed with invasive ductal carcinoma, estrogen receptor, progesterone receptor, E‐cadherin, and Her‐2 positive. She was treated with four cycles of neoadjuvant chemotherapy AC (doxorubicin and cyclophosphamide), then four cycles of THP (docetaxel, trastuzumab, and pertuzumab), and breast wide local excision with axillary clearance followed by radiotherapy and adjuvant therapy, anti her2 blockers (trastuzumab + pertuzumab) for a whole year, and oral aromatase inhibitor (letrozole) that she is still on. She had persistent thrombocytosis before treatment with platelet count range 1011‐1120 × 10^3^/μL. She tested positive for type‐1 calreticulin mutation, and she met the 2016 World Health Organization criteria for ET. She did not have any bleeding or thrombotic complication during and after the treatment for ductal carcinoma. She was started on hydroxyurea for ET with a favorable outcome. Thrombocytosis in patients with cancer is not always reactive but can be linked to MPN, especially ET. Persistent thrombocytosis in these patients needs hematological workup. To the best of our knowledge, this is the first reported case of invasive ductal carcinoma of the breast preceded by ET with type‐1 *CALR* mutation.

Essential thrombocythemia (ET) is a myeloproliferative neoplasm (MPN) characterized by excessive, clonal platelet production.^1^ The diagnosis of ET requires meeting all four major criteria or the first three major criteria and the minor criterion of the World Health Organization (WHO) 2016 for MPN. The major criteria are platelet count ≥450 x 109/L, bone marrow biopsy showing typical findings, not meeting WHO criteria for other BCR‐ABL1+ myeloproliferative neoplasms, and demonstration of a *JAK2*, *CALR*, or *MPL* mutation. The minor criterion is the presence of another clonal marker like (*ASXL1, EZH2, TET2, IDH1/IDH2, SRSF2*, or *SR3B1* mutation) or the absence of evidence for reactive thrombocytosis.^1^ Approximately 90 percent of cases have a somatic mutation in *JAK2*, *CALR*, or *MPL*. *CALR* is a Ca^++^ binding protein primarily located in the endoplasmic reticulum^2^ and functions in the proper folding of newly synthesized glycoproteins and calcium homeostasis.

*CALR* and *JAK2* mutations result in the upregulation of JAK‐STAT {(Janus kinases (*JAK*s), signal transducer and activator of transcription proteins (STATs)}, target genes, which plays a pivotal role in the pathogenesis of ET. Compared with *CALR* mutation, *Jak*2 mutation is more common and carries a worse prognosis and more thrombotic complications.^3^ Additionally, patients with ET are at increased risk of developing another hematological or nonhematological malignancy.^4^ Among solid organ malignancies, breast cancer is the second diagnosed malignancy after lung cancer.^5^ However, breast cancer incidence is not significantly high in patients with myeloproliferative neoplasm relative to the average population.^6^ Nevertheless, coexisting together ET with breast cancer, it is more challenging to manage and predict the outcome of both conditions.

## 2 CASE REPORT

We report a 48‐year post menopause lady with no previous chronic illness, had a family history of breast cancer in two sisters in their forties. Presented with a left breast lump noted during a self‐breast examination. A mammogram performed and a core biopsy of the mass (Left breast, 9‐10 o'clock) showed histopathologic features of invasive ductal carcinoma, SBR grade 2/3. Immunohistochemistry studies with appropriate controls showed: Estrogen receptor‐positive, 2 to 3+ in 90% of cells, progesterone receptor‐positive, 3+ in 90% of cells, and Her‐2 positive (Score 3+), Ki‐67: 70% proliferative index. E‐cadherin: positive. Left axilla and core biopsy: positive for metastatic mammary carcinoma (CK AE1/AE3 immunostain positive). Staging workup, pan CT scan showed bilateral breast masses, enlarged thyroid gland with multiple thyroid nodules, and mall left lung nodule in the lingula, likely metastatic. Bone scan did not show evidence of bone metastasis. The final diagnosis was left breast invasive ductal carcinoma (IDC) T2N1MX suspicious lung nodule ER, PR, and Her2 positive disease. She was started on neoadjuvant chemotherapy AC 4 cycles (started on 7/11/2018), then started THP 4 cycles (14/1/2019 ) + followed by left breast wide local excision and axillary clearance (28/4/2019). She continued her adjuvant treatment after surgery with radiotherapy, targeted therapy anti her2 blockers (trastuzumab and pertuzumab) for a whole year (17 cycles), and hormonal therapy letrozole, and the latter to continue for 5‐10 years.

She tolerated chemotherapy and kept normal left ventricle function throughout the whole year of anti her2 blockers treatment. She felt the breast mass is reducing in size despite that the platelet count was still high. Hematology was involved. A review of her data showed that she has persistent thrombocytosis since the time for breast lump evaluation (platelet count 1011‐1120 × 10^3^/μL, normal range 150‐400 × 10^3^/μL). Workup was done according to the WHO guidelines. Hemoglobin level was13 mg/dl (12‐15 g/dL) and ferritin level was 172 ug/L (12‐240 μg/L). During her clinical course, she had no fever or any sign or symptom indicating active infection. WBC was in the range 4.2‐8.4 × 10^3^/μL normal range (4‐10 × 10^3^/μL), basophils 0.00‐0.1 × 10^3^/μL (normal range 0.02‐0.1 × 10^3^/μL). Peripheral smear shows normochromic normocytic red blood cells and platelets with increased numbers with some large and giant forms. Reticulocytes count was 145.9 × 10^3^/μL (50‐100 × 10^3^/μL). She did not meet WHO criteria for chronic myeloid leukemia (FISH results were normal for BCR/ABL1 t(9;22)(q34;q11.2)).

Further studies showed *Jak*2 was negative,** **calreticulin gene (type‐1 mutation) for a 52bp deletion mutation within exon 9 of the *CALR* gene. She was then scheduled for BM biopsy to rule‐out MPN. Bone marrow biopsy was done, and the results did meet 2016 WHO criteria for chronic myeloid leukemia. Hence, diagnosis with essential thrombocythemia was confirmed.

Further follow‐up with hematology, she was started on hydroxyurea 500 mg daily as cytoreduction on 2.3.2020. Her other medications include aspirin 100 mg, celecoxib 200 mg, daily letrozole 2.5 mg, vitamin D2 50 000 units oral, weekly, and zoledronic acid 4 mg intravenous every 6 months. After starting hydroxyurea on 2/3/2020, platelet count 1132 × 10^3^/μL (150‐400 × 10^3^/μL) after 20 days the follow‐up PLT count improved to 856 × 10^3^/μL (150‐400 × 10^3^/μL).

Until a recent follow‐up, she did not report any thrombotic complications. Her last PET scan after treatment was negative for local and distant disease.

## 3 DISCUSSION

MPNs are a heterogeneous group of disorders characterized by cellular proliferation of one or more hematologic cell lines in the peripheral blood.^7^ They include myelofibrosis, polycythemia vera (PV), and ET. Our patient was diagnosed according to WHO 2016 criteria.^1^ Mutations causing MPN disorders, including ET, can be sporadic or familial.^8^ They carry a significant increase in both hematological and nonhematological malignancies.^4^ Worldwide, breast cancer is the second most frequently diagnosed malignancy, second to lung cancer.^5^ Infiltrating (invasive) ductal carcinoma is the most common type of invasive breast cancer, accounting for around 68.8% of Qatar's breast cancer cases.^9^ The patient was presented initially with a breast lump and managed for invasive ductal carcinoma. ET's diagnosis was delayed: probably because breast cancer‐associated thrombocytosis is uncommon and seen more with inflammatory breast cancer; secondly, it is usually attributed as a paraneoplastic feature that carries a poor prognosis.^10^ Also, breast cancer is not known to be associated with ET. A population base cohort study supports this; previously described breast cancer was not among these solid organ malignancies at these patients with a hazard ratio of 1.4 CI 1.1‐1.1.7 of and standard incidence ratio of 1 of.^6^ However, in this study, the authors described the risk for nonhematological malignancies for MPN patients as a whole with no subgroup risk analysis for PV or ET.

To the best of our knowledge, this is the first patient to be diagnosed with ET with *CALR* mutation type one associated with invasive ductal carcinoma. A review of the literature revealed only one reported case of a patient with ET *Jak*2 mutation associated with infiltrating ductal carcinoma.^11^ ET with *Jak*2 mutation is known to behave more aggressively and carries an unfavorable prognosis than *CALR* mutation.^12^, ^13^ The reported patient with *Jak*2 had more risk factors (*Jak*2 positive, age more than 60 years) but mild platelet count (600 × 10^3^/μL) before starting treatment. Both patients had no report of thrombotic events before, during, or after chemotherapy, noting that platelet count is a poor predictor of thrombosis risk unless more than 1500 × 10^3^/μL as per the International Prognostic Score of thrombosis in World Health Organization essential thrombocythemia (IPSET‐thrombosis score).

**FIGURE 1:**
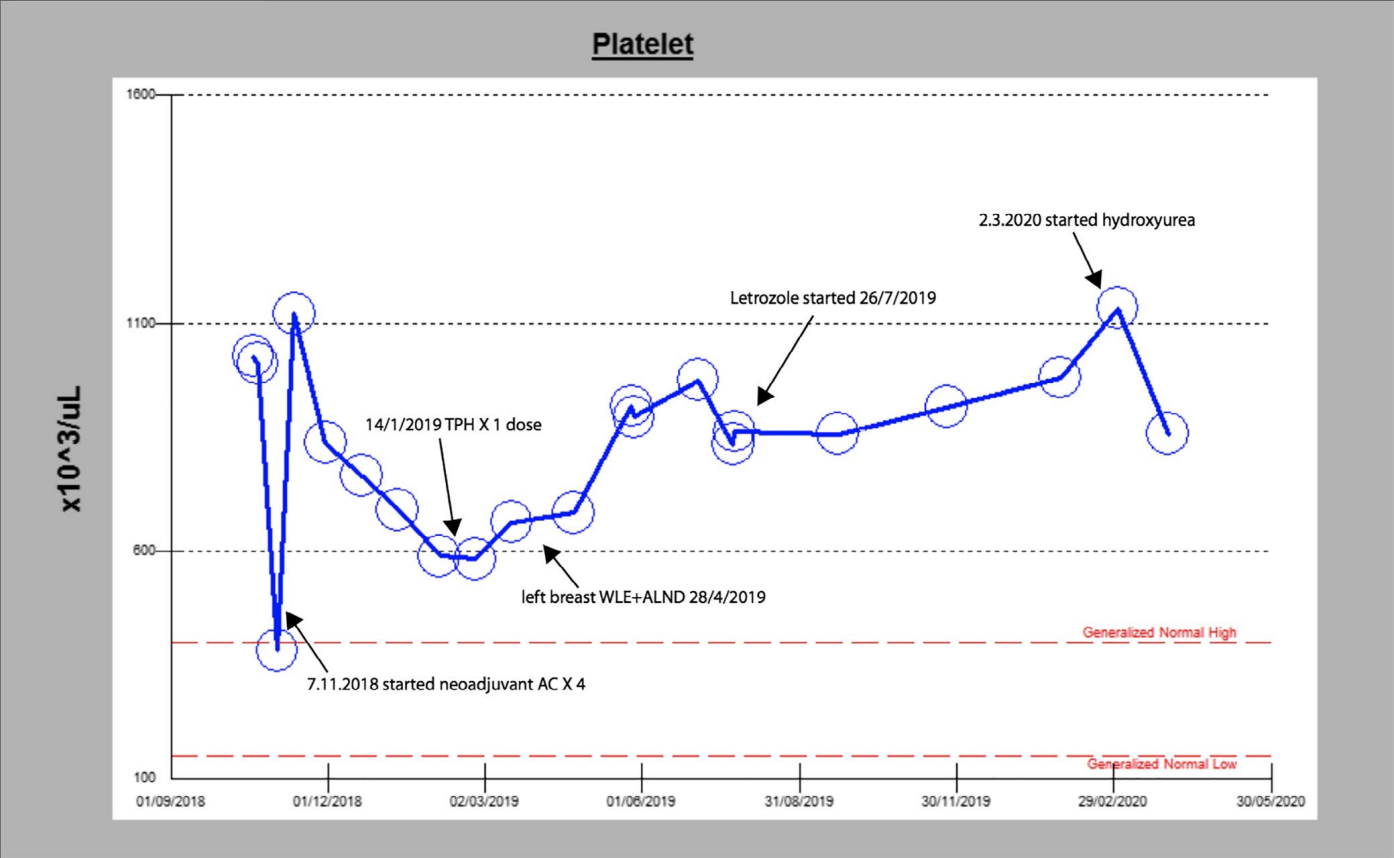
Platelet count from the first presentation until the start of hydroxyurea for 2 months

As thrombocytopenia is a common side effect of breast chemotherapy,^14^ it is not surprising that the patient had neoadjuvant chemotherapy and platelet count dropped significantly, as shown in Figure 1. Chemotherapy‐induced thrombocytopenia may delay the need to start cytotoxic therapy for ET while patients still on chemotherapy, as what happened with this patient and the other patient with *Jak*2 (which was at higher risk). After diagnosis, she was started on hydroxyurea, despite the fact she is at low risk for thrombosis according to (IPSET‐thrombosis).^13^ However, the score is applied to the patient with ET only, not to patients with ET who have another malignancy. Moreover, patients with breast cancer are at risk of thrombosis from the cancer process itself and the hormonal treatment.^15^, ^16^ This makes it reasonable to start these patients on treatment for ET due to the combined risk of thrombosis.

Lwin, Zin‐Mar, et al found that calreticulin protein expressed in breast cancer is correlated with tumor size and metastatic potential.^17^ A similar observation was also noted in gastric and bladder cancer.^18^, ^19^

But mutations in the *CALR* gene leading to breast cancer are not previously described. The normal calreticulin protein is overexpressed in the cancer cells, the mutated gene product of calreticulin may play a major pathological role in the development of breast cancer in these patients.

The question does the ET came first or the breast cancer first is difficult, but most probably, the ET was the first event. The second nonhematological malignancy usually occurs with a mean of 62 months after the diagnosis.^17^ However, occurrence of second malignancy is reported even before the diagnosis of MPN itself, as in this patient might be the case ET running undiagnosed for years. The mechanism is thought to be due to chronic inflammation derived by MPN, which leads to immune deregulation^20^ and impaired “tumor immune surveillance.”

In conclusion, thrombocytosis in patients with breast cancer is uncommon, and its presence should be taken seriously. This is because it is either paraneoplastic, which carries a poor prognosis, or might be a manifestation of serious MPN as ET. Calreticulin is overexpressed in breast cancer cells, and it might be linked to the development of both invasive ductal carcinoma and ET. Management of both conditions should be individualized, and from the observed two patients, breast cancer chemotherapy should not be delayed because of ET; it results in a marked reduction in platelet count without reported hemorrhage or thrombosis as in these two patients. We suggest that these patients be in closer follow‐up because of the multiple risks of thrombosis and ET's unclear effect on breast cancer and vice versa.

## ETHICAL CONSIDERATION

This case was approved by Hamad medical corporation research center, and informed written consent taken from the patient.

## CONFLICT OF INTEREST

All authors have no conflict of interest.

## AUTHORS' CONTRIBUTIONS

Elrazi Ali and Mohamed Yassin: performed writing, editing, and final approval of concept. Nabil E. Omar and Hind Elmalik: performed editing and approval of the final version.

## Data Availability

Data available on reasonable request.
